# First-line pembrolizumab plus chemotherapy for extensive-stage small-cell lung cancer: a United States-based cost-effectiveness analysis

**DOI:** 10.1186/s12962-021-00329-w

**Published:** 2021-12-04

**Authors:** Youwen Zhu, Huabin Hu, Dong Ding, Shuosha Li, Mengting Liao, Yin Shi, Jin Huang

**Affiliations:** 1 Department of Oncology, Xiangya Hospital, Central South University, Changsha, 410008 Hunan China; 2 Department of Medical Oncology, The Sixth Affiliated Hospital of Sun Yat-Sen University, Guangzhou, 510655 China; 3 Guangdong Institute of Gastroenterology, Guangdong Provincial Key Laboratory of Colorectal and Pelvic Floor Diseases, Guangzhou, 510655 China; 4 Department of Health Management Center, Xiangya Hospital, Central South University, Changsha, 410008 Hunan China; 5 Department of Pharmacy, Xiangya Hospital, Central South University, Changsha, 410008 Hunan China; 6 Department of Dermatology, Hunan Engineering Research Center of Skin Health and Disease, Hunan Key Laboratory of Skin Cancer and Psoriasis, Xiangya Hospital, Central South University, Changsha, 410008 Hunan China

**Keywords:** Pembrolizumab, ES-SCLC, Platinum-etoposide, Cost-effectiveness, Quality-adjusted life-years

## Abstract

**Background:**

The clinical trial of Keynote-604 showed that pembrolizumab plus chemotherapy could generate clinical benefits for extensive-stage small-cell lung cancer (ES-SCLC). We aim to assess the efficacy and cost of pembrolizumab combined with chemotherapy in the first-line treatment setting of ES-SCLC from the United States (US) payers’ perspective.

**Methods:**

A synthetical Markov model was used to evaluate cost and effectiveness of pembrolizumab plus platinum-etoposide(EP) versus EP in first-line therapy for ES-SCLC from the data of Keynote-604. Lifetime costs life-years(LYs), quality adjusted LYs(QALYs) and incremental cost-effectiveness ratios(ICERs) were estimated. One-way and probabilistic sensitivity analyses were performed. Furthermore, we performed subgroup analysis.

**Results:**

Pembrolizumab plus EP resulted in additional 0.18 QALYs(0.32 LYs) and corresponding incremental costs $113,625, resulting an ICER of $647,509 per QALY versus EP. The price of pembrolizumab had a significant impact on ICER. Probabilistic sensitivity analysis indicated that pembrolizumab combined chemotherapy may become a cost-effective option with a probability of 0%. Besides, subgroup analysis suggested that all subgroups were not cost-effective.

**Conclusion:**

From the perspective of the US payer, pembrolizumab plus EP is not a cost-effective option for first-line treatment patients with ES-SCLC at a WTP threshold of $150,000 per QALY.

**Supplementary Information:**

The online version contains supplementary material available at 10.1186/s12962-021-00329-w.

## Background

Lung cancer is the main cause of cancer-related death in the United States (US), accounting for more than 13% incidence and 22% mortality in 2021 [1]. Small-cell lung cancer (SCLC) accounts for 10–15% of lung cancers and has a high rate of early metastasis (up to 60–70%) [2, 3]. Overall survival (OS) depends on the initial stage of diagnosis, with a 5-year survival rate of 20–25% for localized-stage SCLC (LS-SCLC) and only 2% for extensive-stage small cell lung cancer (ES-SCLC) [4, 5].

The emergence of immune checkpoint inhibitors (ICIs) has broken the dominant position of etoposide and platinum (EP) regimen in the first-line treatment of ES-SCLC for more than thirty years. ICIs include anti-programmed cell death protein 1 (PD-1) and anti-PD-1 Ligand antibodies (PD-L1), against cytotoxic T-lymphocyte antigen 4 (CTLA-4), some of which have been approved for clinical use [6, 7].

Pembrolizumab is a selective, high-affinity, programmatic, specific, human immunoglobulin G4 monoclonal antibody that binds to the programmed death 1 receptor. It allows anti-tumor T cells to recognize and kill tumor cells [8]. After platinum-based chemotherapy and other therapies failed for advanced SCLC, pembrolizumab monotherapy was approved by the US Food and Drug Administration (FDA) in 2019 [9]. In the first line treatment of patients with ES-SCLC, the randomized phase 3 trial Keynote-604 further demonstrated the efficacy of the combination of pembrolizumab with chemotherapy. It showed that pembrolizumab plus EP therapy prolonged overall survival (OS 10.8 vs 9.7 months; hazard ratio [HR] 0.8; 95% CI 0.64 to 0.98; p = 0.0164) and significantly extended the progression-free survival (PFS 4.5 vs 4.3 months; HR 0.75; 95% CI 0.61 to 0.91; p = 0.0023) compared to EP therapy [10].

However, considering its high cost and limited potential population, economic analysis is urgently needed to evaluate whether a newly approved therapy provides clinical benefit at a justifiable cost and is increasingly necessary to expand its application. Therefore, our goal was to estimate the cost-effectiveness of pembrolizumab plus EP as the first-line treatment of ES-SCLC from the US payers’ perspective.

## Methods

### Model structure

A synthetical Markov model combining a decision tree was established to assess the costs and effectiveness of the different first-line treatment of ES-SCLC for patients, and was similar to the Keynote-604 trial. The decision trees included two treatment: pembrolizumab plus EP group and EP group. The Markov model included three health states to represent the disease course of ES-SCLC: PFS, progressive disease (PD), and death (Additional file 1: Fig. S1 in the electronic supplementary material). All patients in the model started from the PFS state and were treated with pembrolizumab plus EP or EP until disease progression or intolerable toxicity and adverse effects. After PD, patients could receive subsequent treatment, with death as the terminal state. The Markov cycle length in the model was 6 weeks and outcomes were runs for 10 years boundaries. We set the costs and effects of a 3% discount rate per year [11]. Costs, LYs, QALYs, and calculated ICERs were estimated in each treatment regimen. We also considered sub-group cost-effectiveness analysis. The model structure and data were based on results of Keynote-604 [10], the US publicly available databases and published literature [12–15]. The model was constructed viaTreeAge Pro 2020 software (TreeAge Software, Williamstown, MA, https://www.treeage.com).

### Model survival and progression risk estimates

The model operated transition probabilities between health states derived from PFS, OS, and death curves from Keynote-604. We applied the GetData Graph Digitizer (version 2.26; http://www.getdata-graph-digitizer.com/index.php) to collect the data points from the OS and PFS curves and these data points were then used to fit parametric survival models. Then, many parametric survival models were fitted to time-to-event data extracted from Keynote-604 trial, including the Weibull, Exponential, Gompertz, Log-logistic, and Log-normal distributions. According to all aspects, Akaike’s information criterion (AIC) and Bayesian information criterion (BIC) are selected to evaluate the fitting degree of the alternative model. Weibull survival curves which were flexible and widely used were matched to the number of patients in the three states over time, as it can monotonically increase or decrease the hazard function, it is suitable for estimating the event that occurs in the early follow-up work period. The survival model selection was shown in previously published research [16]. the survival model selection was shown in Additional file 1: Table S3 and Fig. S2. Then, we used Weibull distribution to operate in R and we got the two parameters, shape (γ) and scale (λ), were estimated from this fit and applied to Kaplan–Meier curves using the R (version 4.0.2, http://www.r-project.org) and the method proposed by Hoyle and Henley [17].

The time-dependency transition probabilities (tp) are essential in the model analysis. Tp in each Markov cycle was calculated based on the following formula: The Markov cycle is u and the arrival at state t after u Markov cycles is tu was calculated with the following formula: tp(tu) = 1 − exp{λ(t − u)γ − λtγ} (λ > 0, γ > 0) [18].

### Utility estimates

Utility was used to estimate consumer's quality of life (QoL) in the natural history of the disease, on a scale of 0 (death) to 1 (health). We considered the mean health utility score of 0.840 [12] and 0.473 [13] for the PFS and the PD state, respectively. They were based on previously published articles. We also consider the disutility values of 3/4 adverse events (AEs) in our analysis [13–15].

### Cost inputs

We only considered direct costs in 2021 US dollars as follows: the costs of medicines [19, 20], AEs costs (assuming that AEs appeared only one cycle in the PFS and the PD state) [21, 22], costs of laboratory [23] and imaging (every 6 weeks for the first 48 weeks, and every 9 weeks thereafter) [24] and tests administration costs [25] (Table 1 and Additional file 1: Table S1). Based on the Keynote-604 trial, the patients in pembrolizumab plus chemotherapy group: pembrolizumab at a dose of 200 mg once every 3 weeks for 35 cycles and EP [etoposide at a dose of 100 mg/m^2^ on days 1, 2, and 3 and the investigator’s choice of carboplatin (71.10%) at an area under the curve 5 mg/mL per min or cisplatin (28.9%) at a dose of 75 mg/m^2^ every 3 weeks]. The patients in chemotherapy group: EP every 3 weeks for 4 cycles. Patients in the pablizumab plus chemotherapy group received four cycles of induction therapy followed by intravenous injection of 200 mg pablizumab as maintenance therapy. 16.6% patients in the pembrolizumab plus chemotherapy group and 21.1% patients in the chemotherapy group received subsequent anticancer therapy.

**Table 1:** Model parameters: baseline values, ranges, and distributions for sensitivity analysis

Variable	Baseline value	Range		Reference	Distribution
		Minimum	Maximum		
Weibull survival model of OS of PEP Weibull survival model of OS of EP	Scale = 0.035532, Shape = 1.209467 Scale = 0.021012, Shape = 1.497332	– –	– –	[10]	– –
Weibull survival model of PFS of PEP Weibull survival model of PFS of EP	Scale = 0.06893, Shape = 1.40287 Scale = 0.02532, Shape = 2.21179	- –	– –	[10]	– –
Risk for main adverse events in PEP group					
Risk of neutropenia	0.435	0.348	0.522	[10]	Beta
Risk of anemia	0.157	0.126	0.189	[10]	Beta
Risk of thrombocytopenia	0.139	0.111	0.167	[10]	Beta
Risk of leucopenia	0.117	0.094	0.140	[10]	Beta
Risk of pneumonia	0.067	0.054	0.080	[10]	Beta
Risk for main adverse events in EP group					
Risk of neutropenia	0.408	0.326	0.490	[10]	Beta
Risk of anemia	0.152	0.121	0.182	[10]	Beta
Risk of thrombocytopenia	0.112	0.090	0.134	[10]	Beta
Risk of leucopenia	0.094	0.075	0.113	[10]	Beta
Utility					
Utility PFS in first-line treatment	0.840	0.672	1.008	[12]	Beta
Utility PD	0.473	0.378	0.568	[13]	Beta
Disutility due to AEs					
Neutropenia	0.09	0.072	0.108	[13]	Beta
Anemia	0.073	0.058	0.088	[13]	Beta
Leucopenia	0.09	0.072	0.108	[13]	Beta
Pneumonia	0.09	0.072	0.108	[14]	Beta
Thrombocytopenia	0.02	0.016	0.024	[15]	Beta
Drug cost, $/per mg					
Pembrolizumab	20,370.80	16,296.64	24,444.96‬	[20]	Gamma
Etoposide	1,663.73	1,330.98	1996.47	[20]	Gamma
Carboplatin	85.83	68.66	103.00	[20]	Gamma
Cisplatin	51.78	41.42	62.13	[20]	Gamma
Topotecan	199.87	159.90	239.84	[20]	Gamma
Expenditures on main adverse events, $					
Leucopenia	6,831.97	5,465.58	8,198.37	[21]	Gamma
Neutropenia	733.36	586.69	880.03	[22]	Gamma
Anemia	1,199.86	959.89	1,439.83	[22]	Gamma
Thrombocytopenia	851.51	681.21	1,021.82	[22]	Gamma
Pneumonia	5,646.88	4,517.51	6,776.26	[22]	Gamma
Laboratory per cycle	315.14	252.11	378.17	[23]	Gamma
Tumor imaging per cycle	231.10	184.88	167.60	[24]	Gamma
Administration per cycle	139.67	111.74	167.60	[25]	Gamma
Discount rate	0.03	–	–	[11]	–

HR: hazard ratio; OS: overall survival; PFS: progression-free survival; EP: Etoposide and Platinum; PEP: pembrolizumab plus Etoposide and Platinum; AEs: adverse events

The median dosage of medicines was estimated based on standard patients: area under the concentration curve of 5 mg/mL/min and assumed serum creatinine of 1, male sex, 65 years of age, weight of 70 kg, height of 70 inches, and body surface area 1.84 m^2^ [26]. Grade 1/2 events were considered manageable within standard patient monitoring and the correlation with QoL was low [27]. Thus, we included only the cost of managing grade 3/4 AEs (a frequency of greater than 5%) in the model, which had notably different probabilities between the arms of the Keynot-604 trial. All relevant parameters were shown in Table 1.

### Sensitivity analysis

We used a series of sensitivity analyses to predict the uncertainty of the model results. One-way sensitivity analysis was conducted within a variance of 20% from their baseline values according to varied values of a certain parameter within its defined range and the established approaches to examined the individual effects of this parameter on the ICERs (Table 1) [11, 28]. We also conducted probabilistic sensitivity analyses by performing 10,000 Monte Carlo simulations, and the probabilistic sensitivity analysis was completed to assess the variations in multiple parameters at once [29]. A cost-effectiveness acceptability curve of each treatment strategy was evaluated as being the most cost-effective at a certain WTP threshold.

We also considered all patient subgroups of the Keynote-604 trial. In the absence of sufficient data for each patient subgroup, the study adopted the same baseline chemotherapy survival curve for all patients in the chemotherapy group, and their pembrolizumab plus chemotherapy survival curves were produced based on the subgroup-specific HRs according to the approach taken by Hoyle et al. [30] for the absence of OS and PFS curves for each patient subgroup.

## Results

### Base case results

The model projected that life expectancy of patients receiving pembrolizumab plus EP was 1.83 LYs, which was 0.32 LYs more than patients receiving EP. Accounting for QOL, patients receiving pembrolizumab plus EP gained 1.07 QALYs; this value was 0.18 QALYs more than for patients receiving EP. The use of pembrolizumab plus EP cost an additional $113,625, resulting in an ICER of $346,818 per LY, or $647,509 per QALY compared with EP (Table 2).

**Table 2:** Baseline results

Parameters	Pembrolizumab plus EP	EP
LYs	1.83	1.51
QALYs	1.07	0.89
Total cost $	130,692	17,067
ICER $/LY ^a^	346,818	-
ICER $/QALY ^b^	647,509	-
WTP $/QALY	150,000	-

EP: Etoposide and Platinum; ICER: incremental cost-effectiveness ratio; LY: life-year; QALY: quality-adjusted life-year; WTP: willingness-to-pay

^a^ Compared to EP ($/LY)

^b^ Compared to EP ($/QALY)

### Sensitivity analysis

**Fig. 1:**
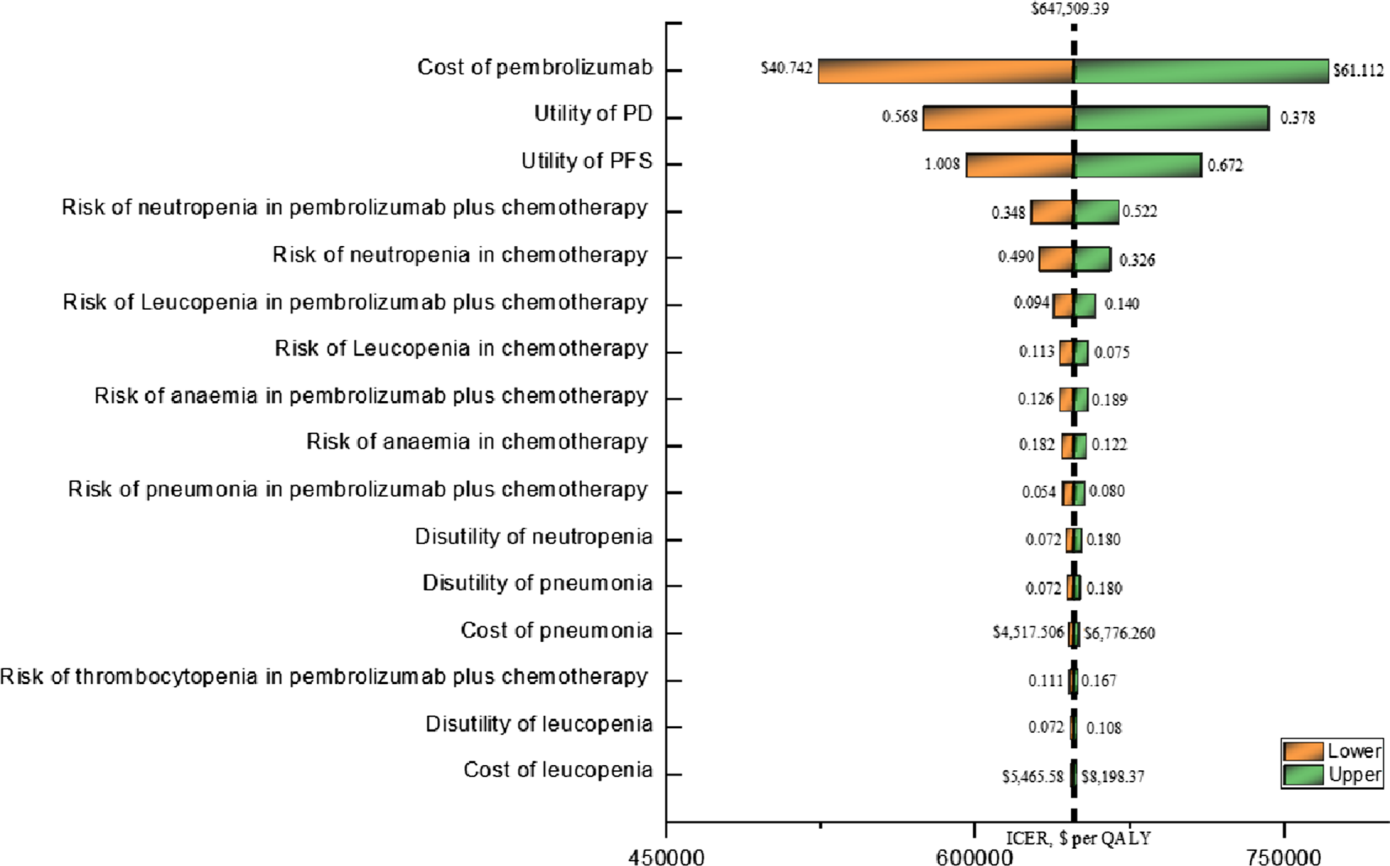
Tornado diagram for one-way sensitivity analysis

One-way sensitivity analysis results (Fig. 1) were most sensitive to the changes in the price of pembrolizumab(ranging from $40.74 to $61.11 per mg, with the ICER increasing from $523,581 per QALY to $771,438 per QALY), followed by the utility of PD, the utility of PFS, the risk of neutropenia in pembrolizumab plus chemotherapy group, the risk of neutropenia in pembrolizumab plus chemotherapy group, and the risk of leucopenia in pembrolizumab plus chemotherapy group.

The ICER scatter diagram (Additional file 1: Fig. S3) showed that the probability sensitivity analysis and pembrolizumab plus EP cannot be effective at the WTP threshold of $150,000 per QALY.

**Fig. 2:**
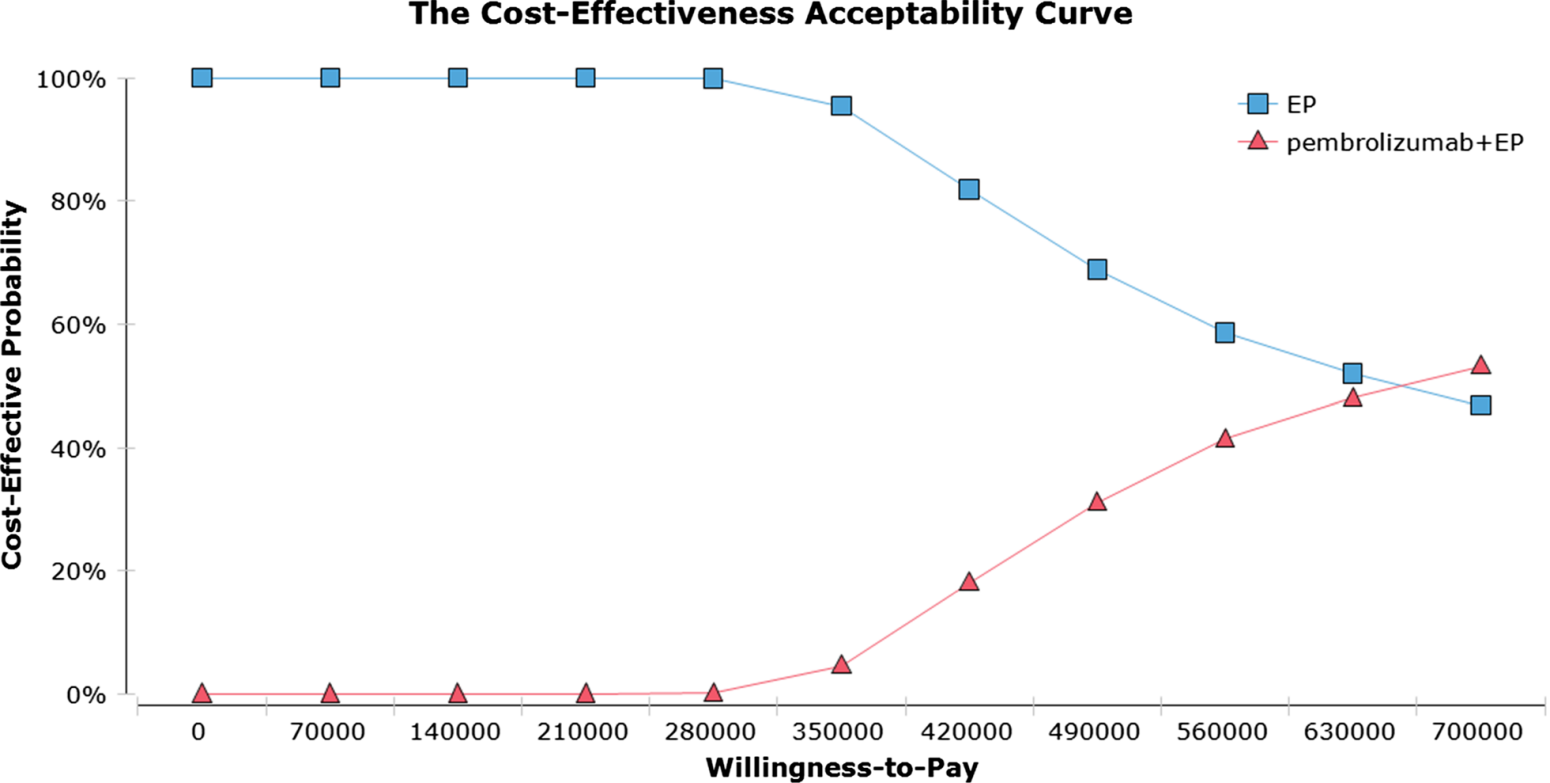
Acceptability curves for the choice of pembrolizumab plus chemotherapy

As shown in the cost-effectiveness acceptability curve(Fig. 2), the probability that the pembrolizumab plus EP strategy is cost-effective increases as the WTP for additional QALY rises. When the cost of pembrolizumab was reduced by 80.3%, and the ICER was $149,904/QALY, which pembrolizumab plus EP was cost-effective. Subgroup analysis declared that the ICER of all patient subgroups was still greater than $150,000/QALY(Additional file 1: Table S2).

## Discussion

In recent years, the emergence of ICIs has greatly changed the treatment strategy of lung cancer, raising a great interest in oncologists and patients**.** There were two studies evaluated the efficacy of ICIs in the first-line treatment of ES-SCLC, Both in CASPIAN [31] and Impower133 [32] trials, first-line immunotherapy plus chemotherapy showed satisfied efficacy. Based on these clinical trials, published research studied the cost-effectiveness of new immunotherapy strategies. According to the data of CASPIAN trial, our published research [33] demonstrated that durvalumab in combination with platinum–etoposide was not a cost-effective option in the first-line treatment of patients with extensive-stage SCLC in the U.S. According to the data of Impower133 trial, Qiu Li [34] and Li [15] conducted cost-effectiveness analysis and concluded that atezolizumab plus chemotherapy to chemotherapy was not a cost-effective choice in the first-line treatment of extensive-stage SCLC from an American and Chinese perspective, respectively. The common point of these studies is that the price of PD-L1 antibody was always the greatest factor affecting the outcomes. Given the updated survival data of the Keynote-604 trial recently published, the cost-effectiveness of pembrolizumab plus chemotherapy in the first-line treatment for patients with ES-SCLC is necessary to be updated accordingly. Therefore, we performed the first cost-effectiveness analysis of pembrolizumab plus chemotherapy versus chemotherapy in first-line setting for patients with ES-SCLC.

Our analysis proved that pembrolizumab plus chemotherapy was not cost-effective in the first-line treatment of ES-SCLC at a WTP threshold of $150,000 per QALY, resulting in an additional 0.18 QALYs and ICER of $ $647,509 per QALY versus chemotherapy. One-way sensitivity analysis showed that the cost of pembrolizumab was the most influential factor. Further, analysis found that when the cost of pembrolizumab was reduced by 80.3%, the immunotherapy became cost-effective at an ICER of $149,904/QALY. Therefore, changing the price of pembrolizumab is an effective feasible strategy to achieve efficient use of pembrolizumab plus chemotherapy. The acceptability curves also demonstrated this finding that a paucity of certainty was achieved by pembrolizumab plus chemotherapy at the WTP threshold of $150,000 in the US. The results of probabilistic sensitivity analysis showed that the probability of pembrolizumab plus chemotherapy was cost-effective vs chemotherapy was 0%. The subgroup cost-effectiveness analysis demonstrated that pembrolizumab plus chemotherapy was not a cost-effective treatment across all patients’ subgroups. Our analysis will be essential to guide policymaking and payment in health care and provided drug pricing decision-makers with the reference to reprice pembrolizumab.

Unfortunately, the detailed expression of PD-L1 and tumor mutational burden (TMB) were not shown in the Keynote-604 trial. In many other studies, PD-L1 expression and TMB were of great value as biomarkers for the efficacy of ICIs treatment, which can improve clinical benefits [35–42]. Anti-PD-1 /PD-L1 antibody is effective in patients with high expression of PD-L1 and high TMB (TMB-H), especially in patients with non-small cell lung cancer (NSCLC), melanoma, renal cell carcinoma, etc. [43, 44]. In our previous studies, we showed that first-line treatment with pembrolizumab was a cost-effective strategy when compared to platinum-based chemotherapy in locally advanced or metastatic NSCLC patients with high expression of PD-L1 (TPS ≥ 50%) [45]. Regardless of the PD-L1 expression levels nivolumab plus ipilimumab was cost-effective in NSCLC patients with TMB-H [46]. It seems that the significance of using predictive markers to properly screen patients and achieve cost-effective strategies is a concern of clinicians and administrators [47]. Therefore, more detailed information about biomarkers is needed in future studies, including PD-L1 expression, TMB, microsatellite instability (MSI) and deficient DNA mismatch repair (dMMR), etc.

Significantly, despite the survival benefits displayed in certain clinical trials, ICIs were not cost-effective in most cases. Consequently, it is a great challenge that to approve new drugs solely based on cost-effectiveness of drugs without considering the dynamic evolution of survival curves and using predictive markers.

There are some limitations in our study. Firstly, our study is based on the Keynote-604 trial, the only clinical trial that estimated the efficacy of pembrolizumab plus EP or EP as first-line therapy in patients with ES-SCLC. Any bias within the trial will be reflected in our study. Secondly, Some of the utility values used in our research are not based on the US, which is indeed an inevitable error. Third,the long-term efficacy of pembrolizumab plus EP in the model was extrapolated from the clinical data from the Keynote-604 trial, which is inevitably subject to uncertainty. Fourth, the Keynote-604 trial did not provide the Kaplan–Meier curve for each subgroup, making it impossible to run the model completely for each subgroup. The original group balance produced by randomization may not exist in the subgroups. Thus, the results of the subgroup analyses should be interpreted with caution. Fifth, we revised the utility value by the disutility of AEs, which will lead to the inaccuracy of the utility value. Sixth, the costs of grade 1/2 AEs and immune-related AEs were excluded, which might overestimate the benefits of pembrolizumab plus chemotherapy. Finally, the compliance of patients was not considered in our study, while a large number of studies have shown that compliance has a significant impact on cost-effectiveness for cancer patients.

## Conclusion

From the perspective of the US payer, pembrolizumab plus EP is not a cost-effective option for first-line treatment patients with ES-SCLC at a WTP threshold of $150,000 per QALY. This finding may help decision-making in healthcare and policy formulation in medical reimbursement.

## Supplementary Information

**Additional file 1: Table S1.** Drug dose and costs. **Table S2.** Results of subgroup analyses. **Table S3.** Summary of statistical goodness-of-fit of K-M curve in Keynote-604 trial. **Figure S1.** Markov states. **Figure S2.** Kaplan–Meier Curve Fitting and Extrapolation. **Figure S3.** Probability Sensitivity Analysis Scatter Plot.

## Data Availability

All authors had full access to all of the data in this study and take complete responsibility for the integrity of the data and accuracy of the data analysis. The datasets generated and/or analyzed during the current study are available from the corresponding author on reasonable request.
