# Supplementary material for: First-line pembrolizumab plus chemotherapy for extensive-stage small-cell lung cancer: a United States-based cost-effectiveness analysis
Source: Cost Eff Resour Alloc. 2021 Dec 4;19:77. doi: 10.1186/s12962-021-00329-w (PMC8642846; doi:10.1186/s12962-021-00329-w)
Supplement: Supplementary file 1 — Additional file 1: Table S1. Drug dose and costs. Table S2. Results of subgroup analyses. Table S3. Summary of statistical goodness-of-fit of K-M curve in Keynote-604 trial. Figure S1. Markov states. Figure S2. Kaplan–Meier Curve Fitting and Extrapolation. Figure S3. Probability Sensitivity Analysis Scatter Plot. [file 12962_2021_329_MOESM1_ESM.docx]

**Figure S1.**Markov states.

**Figure S2.** Kaplan-Meier Curve Fitting and Extrapolation.

**Figure S3.** Probability Sensitivity Analysis Scatter Plot.

**Table S1.** Drug dose and costs.

**Table S2.** Results of subgroup analyses.

**Table S3.** Summary of statistical goodness-of-fit of K-M curve in Keynote-604 trial.

**Figure S1.Markov states.**


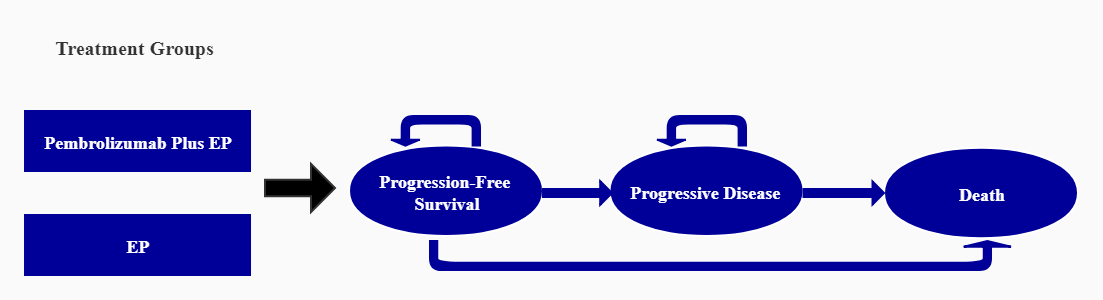


Abbreviation: EP, platinum-etoposide

**Figure S2. Kaplan-Meier Curve Fitting and Extrapolation.**

**
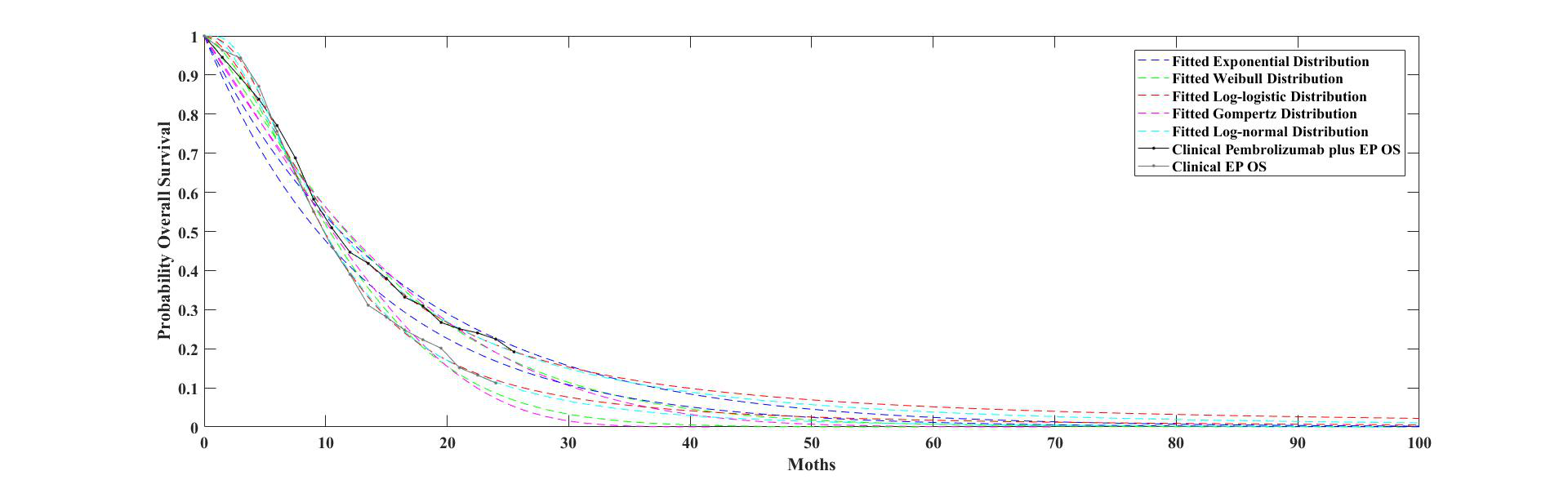
**


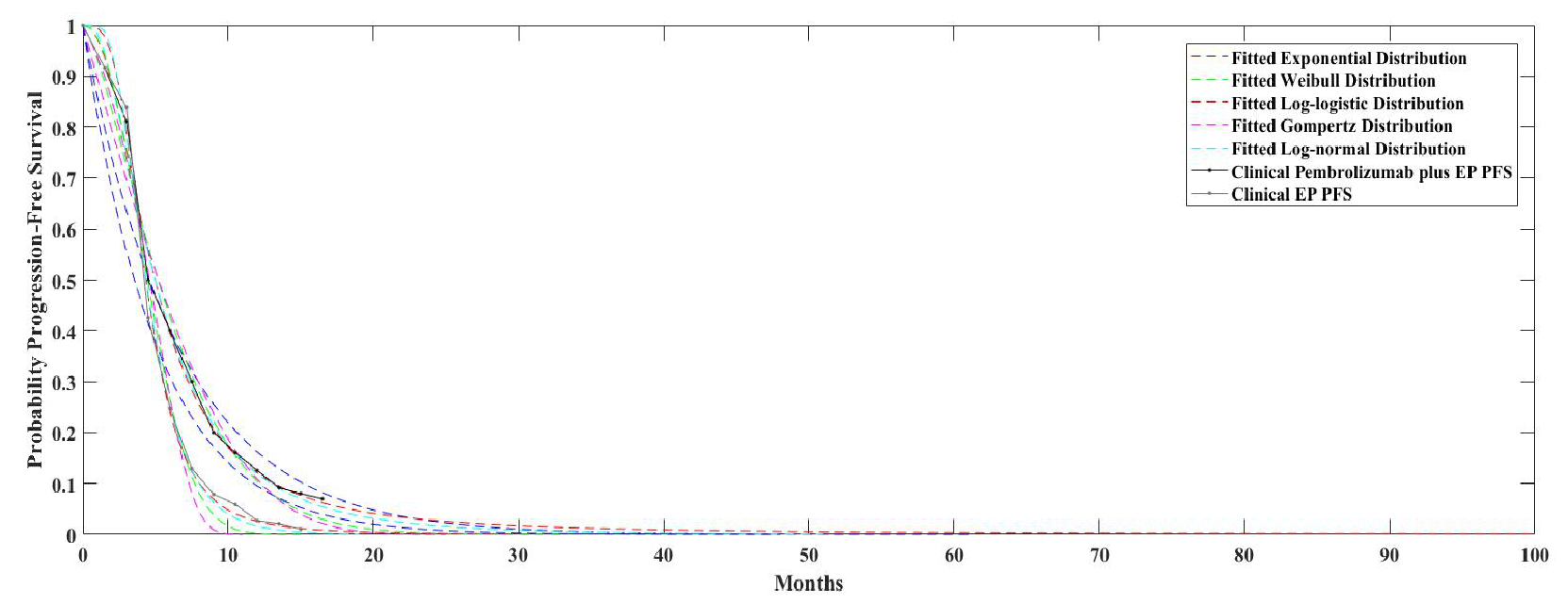
Abbreviation: OS, overall survival; PFS, progression-free survival; EP, platinum-etoposide

**Figure S3. Probability Sensitivity Analysis Scatter Plot.**

**
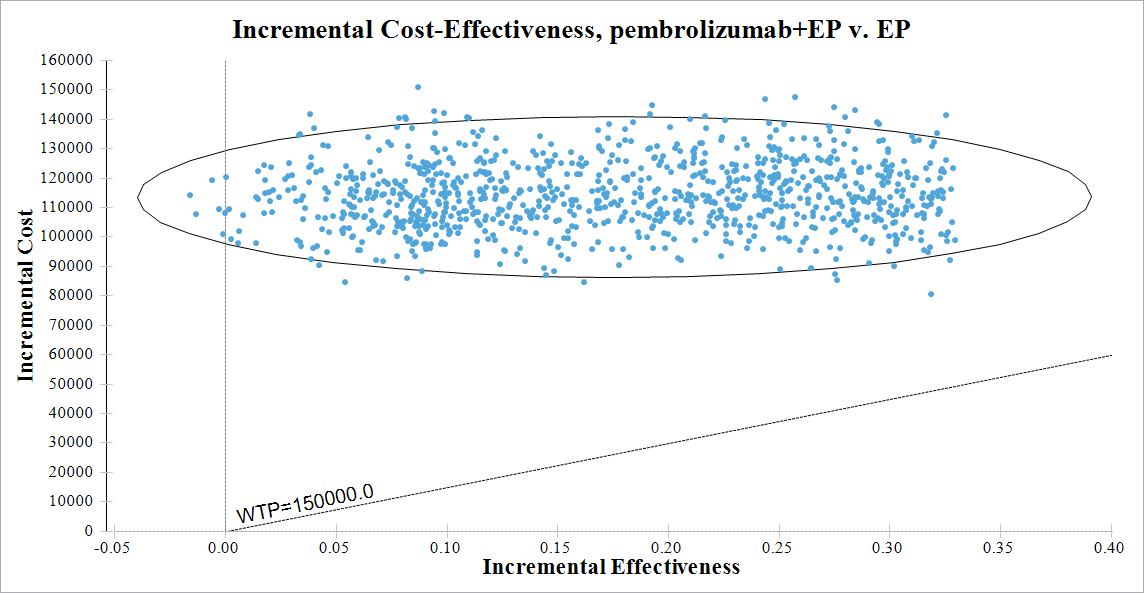
**

Abbreviation: EP, platinum-etoposide; WTP, willingness-to-pay

Each point in the diagram represents a simulation result of 10,000 Monte Carlo simulation. Ellipse represent the 95% CI and dotted line represent WTP ($150,000/QALY). Points to the right of the dotted line are considered cost-effective.

**Table S1. Drug dose and costs**

| **Drug** | **Dose** | **Unit costs(＄)** | **Costs for 1 model cycle (＄, 6 weeks)** |
| --- | --- | --- | --- |
| Pembrolizumab | 200mg every 3 weeks | 50.927 per 1mg | 20370.80 |
| Etoposide | 100 mg/m^2^(on days 1,2,3 For the first 4 cycles) | 1.5071 per 1 mg | 1663.73 |
| Carboplatin | carboplatin area under curve 6(on day 1 of each 3-week cycle) | 0.05772 per 1 mg | 56.52 |
| Cisplatin | 75 mg/m2 (on day 1 of each 3-week cycle) | 0.1876 per 1mg | 51.78 |
| Topotecan | 1.25mg/m2，d1-5 | 8.69 per 1 mg | 199.87 |

**Table S2. Results of subgroup analyses.**

| **Subgroup** | **OS HR (95% CI)** | **PFS HR (95% CI)** | **ICER per QALY (95% CI)** | **WTP** |
| --- | --- | --- | --- | --- |
|  |  |  |  | **$150 000/QALY** |
| **Age, years** |  |  |  |  |
| ＜65 | 0.83(0.61-1.12） | 0.76(0.61- 0.91) | 1,084,639 (425,962 to -1,901,320) | 0% |
| ≥65 | 0.78(0.59-1.05） | 0.70(0.53-0.92） | 818,199 (405,999 to -2,158,192) | 0% |
| **Sex** |  |  |  |  |
| Male | 0.76(0.59 -0.98） | 0.68(0.53-0.87) | 744,207 (405,999 to 9,283,150) | 0% |
| Female | 0.88(0.61-1.26） | 0.78(0.56-1.10） | 1,544,668 (429,759 to -836,958) | 0% |
| **ECOG performance status** |  |  |  |  |
| 0 | 0.68(0.44-1.05） | 0.60(0.40-0.91） | 541,289 (274,615 to -4,266,732) | 0% |
| 1 | 0.86(0.68-1.09） | 0.79(0.63-0.99） | 1,337,166 (540,351 to -2,144,887) | 0% |
|  |  |  |  |  |
| **Region of enrollment** |  |  |  |  |
| East Asia | 0.72(0.44-1.19） | 0.58(0.36-0.94) | 627,172 (280,223 to -1,213,925) | 0% |
| Not East Asia | 0.84(0.67-1.06） | 0.74(0.59-0.92) | 1,143,074 (522,867 to -3,508,986) | 0% |
|  |  |  |  |  |
| **Smoking status** |  |  |  |  |
| Current | 0.86(0.66-1.11） | 0.73(0.57-0.94) | 1,293,212 (506,024 to -1,959,665) | 0% |
| Former | 0.71(0.49-1.02） | 0.72(0.51-1.02) | 604,386 (302,690 to -5,705,962) | 0% |
| **LDH concentration** |  |  |  |  |
| ≤ULN | 0.72(0.52-1.01） | 0.66(0.48-0.91) | 628,498 (333,828 to -17,660,929) | 0% |
| ＞ULN | 0.84(0.65-1.10） | 0.66(0.48-0.91) | 1,153,023 (488,617 to -1,997,010) | 0% |
| **No. of metastatic sites** |  |  |  |  |
| ＜3 | 1.04(0.72-1.50） | 0.70(0.50 -0.97) | -34,568,331 (625,735 to -533,370) | 0% |
| ≥3 | 0.71(0.55-0.92) | 0.76(0.59-0.97) | 604,464 (353,700 to 2,871,003) | 0% |
| **Baseline brain metastasis** |  |  |  |  |
| Yes | 1.32(0.72-2.42) | 1.07(0.60-1.91) | -705,738 (627,512 to -189,227) | 0% |
| No | 0.75(0.60-0.94) | 0.69(0.55-0.85) | 713,090 (417,130 to 3,221,423) | 0% |
| **Baseline liver metastasis** |  |  |  |  |
| Yes | 0.75(0.55-1.02) | 0.90(0.67-1.21) | 726,881 (346,890 to -3,418,128) | 0% |
| No | 0.82(0.62-1.08) | 0.64(0.49-0.83) | 982,126 (449,233 to -3,354,969) | 0% |
| **PD-L1 CPS** |  |  |  |  |
| ＜1 | 0.80(0.58-1.11) | 0.73(0.54-1.01） | 909,469 (392,758 to -1,766,446) | 0% |
| ≥1 | 0.84(0.60-1.18） | 0.68(0.49-0.94） | 1,113,383 (422,343 to -1,272,239) | 0% |
| **Platinum administered** |  |  |  |  |
| Cisplatin | 0.73(0.49-1.08） | 0.60(0.41-0.88） | 652,301 (314,494 to -2,954,091) | 0% |
| Carboplatin | 0.83(0.65-1.07） | 0.77(0.60-0.97） | 1,088,752 (487,506 to -2,737,006) | 0% |

Abbreviation: CI, confidence interval; ICER, incremental cost-effectiveness ratio; LY, life-year; OS HR, overall survival hazard ratio; PFS HR, progression-free survival hazard ratio; LDH, lactate dehydrogenase; ULN, upper limit of normal; PD-L1,programmed cell death-Ligand 1; CPS, combined positive score; QALY, quality-adjusted life-year; WTP, Willingness-to-pay.

The results of subgroup analyses implied that the ICER remained greater than $150,000/QALY in all patient subgroups. The results of subgroup probabilistic sensitivity analyses suggested that all subgroups were not cost-effective.

**Table S3. Summary of statistical goodness-of-fit of K-M curve in Keynote-604 trial.**

| **Distribution** | **Pembrolizumab plus EP OS** | | **EP OS** | | **Pembrolizumab plus EP PFS** | | **EP PFS** | |
| --- | --- | --- | --- | --- | --- | --- | --- | --- |
|  | **AIC** | **BIC** | **AIC** | **BIC** | **AIC** | **BIC** | **AIC** | **BIC** |
| Exponential Distribution | 30.41 | 31.30 | 32.31 | 33.14 | 34.30 | 34.70 | 32.00 | 32.49 |
| Weibull Distribution | 30.28 | 30.06 | 31.87 | 32.54 | 33.35 | 33.15 | 31.80 | 31.77 |
| Gompertz Distribution | 33.24 | 35.02 | 38.26 | 39.93 | 240.05 | 240.85 | 40.55 | 41.52 |
| Log-logistic Distribution | 32.83 | 34.61 | 36.63 | 38.29 | 49.19 | 49.99 | 36.70 | 37.67 |
| Log-normal Distribution | 32.76 | 34.55 | 36.67 | 38.33 | 53.88 | 54.67 | 37.12 | 38.10 |

Abbreviation: EP, etoposide and platinum; OS, overall survival; PFS, Progression-free survival; AIC, Akaike’s information criterion; BIC, Bayesian information criterion.

As for the ten curves listed in the table, the Weibull distribution had the lowest AIC and BIC. Weibull distributions are flexible and wildly used in cancer survival analyses. Therefore, the Weibull distributions was likely to be the most reasonable parametric survival model.
